# WORKING LONGER FOR ACADEMICS IN THE U.S. AND IRELAND: A GENDERED LIFECOURSE ANALYSIS

**DOI:** 10.1093/geroni/igz038.469

**Published:** 2019-11-08

**Authors:** Aine Ni Leime

**Affiliations:** National University of Ireland, Galway Ireland, Galway, Ireland, Ireland

## Abstract

Many governments including the US and Ireland have been advocating longer working lives for all workers to ensure pension sustainability in the light of population ageing. Policy changes encouraging increased social security/state pensions age reflect this. However, there has been limited investigation of how the gender implications of these policy changes. While longer working lives may be attractive for some workers, there is evidence that women and men have profoundly different work-life trajectories and women may be more financially disadvantaged approaching retirement age. There is a need to explore how this affects their ability and/or desire to continue working past traditional retirement age and their financial security. This presentation is based on analysis of evidence from an EU-funded cross-national research project involving work-life history interviews conducted with forty older workers in academia in the US and Ireland in 2016 and 2017. A lifecourse approach is used to analyse interview data from ten male and ten female academics in Ireland and ten male and ten female academics in the US, aged 50 or over. Participants discussed early influences, work-life history and health concerns. The paper uses a cumulative disadvantage perspective to analyse how gender, family and health trajectories across the life course affect and can limit options around late work and retirement. It concludes that gender differences regarding norms of care-giving are important and that extending working life is more likely to be caused by financial necessity for women. The implications for future research and policy are discussed.

